# Supplementary material for: Aberrant hippocampal Ca2+ microwaves following synapsin-dependent adeno-associated viral expression of Ca2+ indicators
Source: eLife. 2024 Jul 23;13:RP93804. doi: 10.7554/eLife.93804 (PMC11265795; doi:10.7554/eLife.93804)
Supplement: MDAR checklist [file elife-93804-mdarchecklist1.pdf]

eLife

Westbrook Centre
Milton Road
Cambridge CB4 1YG, UK

P 01223 855340
W elifesciences.org
T @elife

Materials Design Analysis Reporting (MDAR) Checklist for Authors

The MDAR framework establishes a minimum set of requirements in transparent reporting mainly applicable to studies in the life sciences.

eLife asks authors to **provide detailed information within their article** to facilitate the interpretation and replication of their work. Authors can also upload supporting materials to comply with relevant reporting guidelines for health-related research (see EQUATOR Network), life science research (see the BioSharing Information Resource), or animal research (see the ARRIVE Guidelines and the STRANGE Framework; for details, see *eLife*'s Journal Policies). Where applicable, authors should refer to any relevant reporting standards materials in this form.

For all that apply, please note **where in the article** the information is provided. Please note that we also collect information about data availability and ethics in the submission form.

Materials:

Newly created materials	Indicate where provided: section/figure legend	N/A
The manuscript includes a dedicated "materials availability statement" providing transparent disclosure about availability of newly created materials including details on how materials can be accessed and describing any restrictions on access.	Patent application entitled “Peptides targeting sodium channels to treat pain” based on this research was filed by the University of California. In accordance with the guidelines about the sharing of unique research resources, we will make the designed peptides available to the scientific community after protecting IP (the designed peptide sequences and structures). Requests from for-profit corporations will be negotiated by the Office of Technology Management of University of California, Davis. All licensing shall be subject to distribution pursuant to University of California, Davis policies and procedures on royalty income.	N/A

Antibodies	Indicate where provided: section/figure legend	N/A
For commercial reagents, provide supplier name, catalogue number and RRID, if available.	see the Key resources table	N/A

DNA and RNA sequences	Indicate where provided: section/figure legend	N/A
Short novel DNA or RNA including primers, probes: Sequences should be included or deposited in a public repository.	see the Key resources table	N/A

Cell materials	Indicate where provided: section/figure legend	N/A
Cell lines: Provide species information, strain. Provide accession number in repository OR supplier name, catalog number, clone number, OR RRID.	Tsa201 cell line from Dr. William Catterall (University of Washington).<br>Human Nav1.7 cell line from Dr. Christoph Lossin (University of California, Davis).<br>Human Nav1.8 and Nav1.9 stable cell lines from Dr. Neil Castle (Icagen).<br>Human Nav1.2 cDNA from Dr. Alan L. Goldin (University of California, Irvine).	N/A
Primary cultures: Provide species, strain, sex of origin, genetic modification status.		N/A

Experimental animals	Indicate where provided: section/figure legend	N/A
Laboratory animals or Model organisms: Provide species, strain, sex, age, genetic modification status. Provide accession number in repository OR supplier name, catalog number, clone number, OR RRID.	MrgprDGFP mouse line from Dr. David Ginty (Harvard University).<br>see animals section of methods	
Animal observed in or captured from the field: Provide species, sex, and age where possible.		N/A

Plants and microbes	Indicate where provided: section/figure legend	N/A
Plants: provide species and strain, ecotype and cultivar where relevant, unique accession number if available, and source (including location for collected wild specimens).		N/A
Microbes: provide species and strain, unique accession number if available, and source.		N/A

Human research participants	Indicate where provided: section/figure legend) or state if these demographics were not collected	N/A
If collected and within the bounds of privacy constraints report on age, sex, gender and ethnicity for all study participants.		N/A

Design:

Study protocol	Indicate where provided: section/figure legend	N/A
If the study protocol has been pre-registered, provide DOI. For clinical trials, provide the trial registration number OR cite DOI.		N/A

Laboratory protocol	Indicate where provided: section/figure legend	N/A
Provide DOI OR other citation details if detailed step-by-step protocols are available.		N/A

Experimental study design (statistics details) *		
**For in vivo studies: State whether and how the following have been done**	**Indicate where provided: section/figure legend. If it could have been done, but was not, write "not done"**	**N/A**
Sample size determination	Yes - see the Methods section entitled "Statistical analysis".	Not Done
Randomisation	Yes - see the Methods section entitled "Statistical analysis".	Not Done
Blinding	Yes - see the Methods section entitled "Statistical analysis".	Not Done
Inclusion/exclusion criteria	Yes - see the Methods section entitled "Statistical analysis".	NA

Sample definition and in-laboratory replication	Indicate where provided: section/figure legend	N/A
State number of times the experiment was replicated in the laboratory.	Experiments were replicated at least 3 times. Sample sizes are shown in Tables and Figure legends, and represent biological replicates (individual cells or animals).	see Fig 2

Define whether data describe technical or biological replicates.	Data describe biological replicates (individual cells or animals).	

**Ethics**	**Indicate where provided: section/submission form**	**N/A**
Studies involving human participants: State details of authority granting ethics approval (IRB or equivalent committee(s), provide reference number for approval.		N/A
Studies involving experimental animals: State details of authority granting ethics approval (IRB or equivalent committee(s), provide reference number for approval.	Research involving vertebrate animals was done at the University of California following protocols reviewed and approved by the UC Davis Institutional Animal Care and Use Committee (UCD IACUC) - Animal Welfare Assurance Number A3433-01. The animals were cared for by the Center for Laboratory Animal Science (CLAS) Veterinary Services under a currently AAALAC approved program under the direction of Dr. Laura Brignolo (Campus Veterinarian). The animals were housed in NIH-approved facilities in CLAS and are observed daily by technicians. Unusual events are reported to the on call veterinarian, as well as to the investigator according to posted protocols. Other maintenance veterinary care was conducted according to NIH guidelines on the Use and Care of Animals. Facilities were inspected regularly according to NIH and AAALAC guidelines.	see methods. Animal section
Studies involving specimen and field samples: State if relevant permits obtained, provide details of authority approving study; if none were required, explain why.		N/A

**Dual Use Research of Concern (DURC)**	**Indicate where provided: section/submission form**	**N/A**
If study is subject to dual use research of concern regulations, state the authority granting approval and reference number for the regulatory approval.		N/A

Analysis:

Attrition	Indicate where provided: section/figure legend	N/A
Describe whether exclusion criteria were pre-established. Report if sample or data points were omitted from analysis. If yes, report if this was due to attrition or intentional exclusion and provide justification.	All data generated or analyzed are included in the figures.<br>correct. all data analysed	

Statistics	Indicate where provided: section/figure legend	N/A
Describe statistical tests used and justify choice of tests.	see results each individual comparison described	

Data availability	Indicate where provided: section/submission form	N/A
For newly created and reused datasets, the manuscript includes a data availability statement that provides details for access (or notes restrictions on access).	All data associated with this study are present in the paper or the Supplementary Materials.	
When newly created datasets are publicly available, provide accession number in repository OR DOI and licensing details where available.	Many example videos are included in the manuscript and supporting files; due to size restrictions the full videos are available upon request. Data and code required to reproduce the summary data figures is available at (https://github.com/tonykelly00/Ca_waves_data-Elife).	N/A
If reused data is publicly available provide accession number in repository OR DOI, OR URL, OR citation.	Protein st[illegible] human Na[illegible] chimera i[illegible] II used in [illegible] download[illegible] Bank: https://w[illegible] N4R	NA

Code availability	Indicate [illegible] section/f[illegible]	N/A
For any computer code/software/mathematical algorithms essential for replicating the main findings of the study, whether newly generated or re-used, the manuscript includes a data availability statement that provides details for access or notes restrictions.	Rosetta software is publicly available at: https://www.rosettacommons.org/software/license-and-download	NA
Where newly generated code is publicly available, provide accession number in repository, OR DOI OR URL and licensing details where available. State any restrictions on code availability or accessibility.		N/A

If reused code is publicly available provide accession number in repository OR DOI OR URL, OR citation.		N/A

Reporting:

The MDAR framework recommends adoption of discipline-specific guidelines, established and endorsed through community initiatives.

**Adherence to community standards**	**Indicate where provided: section/figure legend**	**N/A**
State if relevant guidelines (e.g., ICMJE, MIBBI, ARRIVE, STRANGE) have been followed, and whether a checklist (e.g., CONSORT, PRISMA, ARRIVE) is provided with the manuscript.		N/A

---

* We provide the following guidance regarding transparent reporting and statistics; we also refer authors to [Ten common statistical mistakes to watch out for when writing or reviewing a manuscript](#).

Sample-size estimation

- You should state whether an appropriate sample size was computed when the study was being designed
- You should state the statistical method of sample size computation and any required assumptions
- If no explicit power analysis was used, you should describe how you decided what sample (replicate) size (number) to use

Replicates

- You should report how often each experiment was performed
- You should include a definition of biological versus technical replication
- The data obtained should be provided and sufficient information should be provided to indicate the number of independent biological and/or technical replicates
- If you encountered any outliers, you should describe how these were handled
- Criteria for exclusion/inclusion of data should be clearly stated
- High-throughput sequence data should be uploaded before submission, with a private link for reviewers provided (these are available from both GEO and ArrayExpress)

Statistical reporting

- Statistical analysis methods should be described and justified
- Raw data should be presented in figures whenever informative to do so (typically when N per group is less than 10)
- For each experiment, you should identify the statistical tests used, exact values of N, definitions of center, methods of multiple test correction, and dispersion and precision measures (e.g., mean, median, SD, SEM, confidence intervals; and, for the major substantive results, a measure of effect size (e.g., Pearson's r, Cohen's d)
- Report exact p-values wherever possible alongside the summary statistics and 95% confidence intervals. These should be reported for all key questions and not only when the p-value is less than 0.05.

Group allocation

- Indicate how samples were allocated into experimental groups (in the case of clinical studies,

please specify allocation to treatment method); if randomization was used, please also state if restricted randomization was applied

- Indicate if masking was used during group allocation, data collection and/or data analysis
